# Supplementary material for: A comprehensive re-assessment of the association between vitamin D and cancer susceptibility using Mendelian randomization
Source: Nat Commun. 2021 Jan 11;12:246. doi: 10.1038/s41467-020-20368-w (PMC7801600; doi:10.1038/s41467-020-20368-w)
Supplement: Supplementary file 1 — Supplementary Information [file 41467_2020_20368_MOESM1_ESM.pdf]

Supplementary materials for the manuscript

A comprehensive re-assessment of the association between vitamin D and cancer susceptibility using Mendelian randomization

Table of Contents

Supplementary methods 3

Deriving the proportion of variance in 25(OH)D tagged by SNP instruments 3

Power calculations for Mendelian randomization analyses 3

Estimating the between-sex genetic correlation for serum 25(OH)D levels 3

Description of the QSkin cohort 3

Description of the 23andMe cohort 4

Source of GWAS summary statistics for each cancer 4

Literature search for previous published MR findings for 25(OH)D and individual cancers 5

Description of alternative MR causal effect estimators applied in the MR analysis 5

Preparation of GWAS on sun-exposure and pigmentation phenotypes for multivariable MR analysis 6

Supplementary Tables 8

Supplementary Table 1. Comparison of the proportion of 25(OH)D variance explained by SNP instruments estimated using the UKB cohort 8

Supplementary Table 2. Estimation of power for Mendelian randomization analyses for various cancers based on total sample size and proportion of phenotypic variance explained by 25(OH)D SNP instruments 9

Supplementary Table 3. MR association between 25(OH)D and skin cancers excluding 25(OH)D variants* associated with skin colour, facial aging and episodes of childhood sunburn 10

Supplementary Table 4. Reverse MR findings for changes in 25(OH)D concentration per genetically predicted doubling of odds on cancer risk. 11

Supplementary Table 5. MR association between per SD increase in 25(OH)D and cancer risk using sex-specific 25(OH)D genetic instruments. 11

Supplementary Table 6. Comparison of MR association between one SD increase in 25(OH)D concentration and risk of cancer(s) with/without adjustment for vitamin D supplementation use. 12

Supplementary Table 7. MR association between 25(OH)D and cancer risk under natural log(25(OH)D) and rank-transformed 25(OH)D scales. 13

Supplementary Table 8. Comparison of log(25(OH)D) effect estimates for 25(OH)D instruments reported in various studies. 16

Supplementary Table 9. Evaluation of instrument strength for the multivariable MR analysis adjusting for pigmentation related variables via the Sanderson-Windmeijer conditional F-statistic. 17
Supplementary Table 10. Multivariable MR association between 25(OH)D and skin cancers adjusted for pigmentation related variables using the original 8 candidate traits 18
Supplementary Figures 20
Supplementary Figure 1. Scatter plot for the MR association between 25(OH)D and breast cancer risk.. 21
Supplementary Figure 2. Scatter plot for the MR association between 25(OH)D and endometrial cancer risk. 22
Supplementary Figure 3. Scatter plot for the MR association between 25(OH)D and EOC risk. 23
Supplementary Figure 4. Scatter plot for the MR association between 25(OH)D and prostate cancer risk. 25
Supplementary Figure 5. Scatter plot for the MR association between 25(OH)D and the risk of BE and EA combined (BEEA). 26
Supplementary Figure 6. Scatter plot for the MR association between 25(OH)D and risk of skin cancers. 27
Supplementary Figure 7. Funnel plots for the MR associations between 25(OH)D and cancer risk. 28
Supplementary Figure 8. Sketch diagram illustrating potential inflation of MR OR due to ascertainment bias on history of skin disorder for the MR findings on vitamin D and skin cancer risk. 30
References 31
Supplementary Note 33
The PRACTICAL consortium 33
The BCAC consortium 34
The OCAC consortium 34
The ECAC consortium 34
The QSkin Genetic study 35
The GenoMEL consortium 35
The Esophageal Cancer consortium (BEACON, CAMBRIDGE, OXFORD and the BONN study) 35

Supplementary methods

Deriving the proportion of variance in 25(OH)D tagged by SNP instruments

The proportion of variance on the exposure (X) explained by SNP instruments, denoted as r_{total}^2 can be derived using:

$$r_{total}^2 = \frac{\sum_{i=1}^{m} 2(p_i)(1-p_i)\beta_i^2}{Var(X)}$$

for m independent 25(OH)D SNP instruments where p_i refer to the effect allele frequency of SNP_i and β_i refer to magnitude of association between SNP_i with X.

Power calculations for Mendelian randomization analyses

We estimated the statistical power to detect an association between a one SD change in genetically predicted 25(OH)D and cancer risk at various pragmatic cut-offs (OR>1.1, OR>1.2 and OR>1.4) using the mRnd (http://cnsgenomics.com/shiny/mRnd/) online MR power calculator [1]. We set the proportion of phenotypic variance (25(OH)D) explained by SNPs to be 4.5%. The estimated power for each of the cancer MR analyses were shown in Supplementary Table 2.

Estimating the between-sex genetic correlation for serum 25(OH)D levels

Sex-specific genetic markers can potentially yield more accurate causal estimates for MR if the SNP-exposure association differs between sexes. We first evaluated the genetic correlation on 25(OH)D between white British men and women in the UKB through linkage disequilibrium (LD) score regression [2]using the GWAS summary statistics on rank-transformed 25(OH)D concentration performed separately for men and women. The data cleaning and curation of both of these GWAS datasets were similar to the approach adopted in the main text other than individuals of the opposing sex being excluded (rather than fitting sex as a covariate, all women were excluded from the male-specific GWAS, vice versa). The LD-score derived genetic correlation is 0.9599 (se: 0.0287) indicating substantially high genetic overlap between 25(OH)D in men and women providing poor rationale for the use of sex-specific instrument. Hence, we proceeded our MR analyses using only the 25(OH)D GWAS performed on both sex combined (genetic sex only fitted as a covariate in the GWAS model) in the UKB to maximise power for instrument detection.

Description of the QSkin cohort

The QSkin cohort [3] is a large Australian-based prospective study on skin cancers. The cohort consists of 43 794 young to middle aged (40-69) adults, the majority being of white European ancestry. The primary aim of the study is to identify and evaluate both environmental and

genetic risk factors associated with skin cancer. Data on each participant's phenotype, lifestyle, behaviour and exposures to environmental risk factors were obtained through self-report, in 2011. Keratinocyte cancers (KC) are not routinely registered in the Australian cancer registries, hence health administration data obtained from the Australian Medicare database were used to identify individuals that underwent treatments for KCs for the period upon date of consent through to June 30, 2014. Exact diagnosis of KCs (BCC and SCC) were established through further linkage with medical (pathology) records. The complete description of the cohort profile for QSkin can be found elsewhere [3].

Description of the 23andMe cohort

The 23andMe company [4] is a personalised genomics USA-based corporation, with its headquarters situated in Mountain View, California, USA. The company has a huge customer base that consented for participation in research. As per GWAS analyses in Liyanage et al. [5], 23andMe GWAS summary data on SCC and BCC [6,7] were derived from 23andMe participants who answered the self-reported questionnaire on whether they had a history of SCC/BCC diagnosis. The accuracy of the self-reported SCC and BCC status were verified clinically through a separate clinical study where Chahal et al. [7] showed good concordance between the self-report data and medical records. Genotyping for the 23andMe participants was conducted using several different customised arrays, which included custom variants and variants tagged from the Illumina HumanHap550+ BeadChip, and Illumina OmniExpress+ BeadChip arrays [6].

Source of GWAS summary statistics for each cancer

The GWAS summary statistics for breast cancer risk reported in Michailidou et al. [5,8] was obtained from the official BCAC online repository, available here: http://bcac.ccge.medschl.cam.ac.uk/bcacdata/oncoarray/. The GWAS summary statistics for the prostate cancer risk reported in Schumacher et al. [9] was obtained from the official BCAC online repository, available here: http://practical.icr.ac.uk/blog/?page_id=8164. The GWAS data accompanying the work by Phelan et al. [10] is publicly available and can be obtained through written request to the OCAC program committee (http://ocac.ccge.medschl.cam.ac.uk/). The ILCCO GWAS for lung cancer [11], as well as the genetic summary data on neuroblastoma and pancreatic cancer (PanScan) were already deposited in the MR-Base database and we analysed these data directly through the MR-web based interface (http://app.mrbase.org/). Genetic summary data for melanoma (GenoMEL) [12] and esophageal cancers (BEACON) [13] were provided through formal application for use of the data through the respective program committee for each consortium. Data for the SCC and BCC GWAS meta-analysis can be obtained from written request to Dr. Stuart MacGregor (email: stuart.macgregor@qimrberghofer.edu.au), excluding the 23andMe data.

Literature search for previous published MR findings for 25(OH)D and individual cancers

We performed a literature search on the PUBMED/PUBMED Central database published articles detailing MR findings between 25(OH)D concentration and cancer risks for cancers evaluated in our present analysis. The following keywords were used in combinations: "Mendelian randomization", "Mendelian randomisation", "Cancer risk", "Cancers", "Melanoma", "Keratinoctye cancers", "Vitamin D", "25(OH)D", "genetically predicted vitamin D", "causal inference", "instrumental variable". Publications for each individual type of cancer were then filtered for the following criteria: (i) total sample size exceeds 2000. (ii) Reported effect estimates for quantifiable changes in 25(OH)D concentration (i.e. nmol/L, log(nmol/L) or SD units). (iii) Analyses based on European participants. For individuals with multiple published MR findings, we prioritise to select only the study with the largest sample size.

Description of alternative MR causal effect estimators applied in the MR analysis

Weighted median estimator

The weighted median MR estimator [14] enables consistent estimation of the MR causal effect even when close to 50% of instruments are invalid instruments. The penalised weighted median model is a slight modification of the weighted median estimator by down-weighting SNPs that are highly heterogeneous ratio estimates.

Weighted mode estimator

In contrast to the median-based estimators, the mode based estimators [15] does not require more than 50% of the instruments being valid. Instead, the mode based estimators yield consistent estimates even when majority instruments are invalid instruments by relying on plurality such that ratio estimands from the largest subset of variants with similar effect sizes (largest group) constitute the true estimate. The weighted mode estimator assumes that the most frequent ratio estimand across all instrument is zero (also known as the Zero Modal Pleiotropy Assumption, ZEMPA).

MR-Egger

MR-Egger regression help detect the presence of directional pleiotropy in IVW MR findings (captured by the MR-Egger intercept) and provide less biased estimates of the causal effect. The method relies on the InSIDE assumption [16] being valid.

MR-PRESSO

MR-PRESSO [17] is a leave-one-out based approach that evaluates the extent of horizontal pleiotropy bias and heterogeneity in MR causal estimates.The approach can be summarised in three steps. First, MR-PRESSO performs a global test to evaluate whether the total residual sums of squares (RSS) (which was computed by leaving one SNP out in turn) is consistent with those expected by chance. The model then performs an outlier test by utilizing the RSS of individual SNPs to identify outliers. The final stage incorporates a distortion test to evaluate

which identified outlier meaningfully changed the MR causal estimates. We used the following default setting for each trait-pair analysed: number of iterations=4000, distortion_test=TRUE, outlier_test=TRUE, alpha=0.05. The MR-PRESSO software can be readily downloaded from https://github.com/rondolab/MR-PRESSO.

IVW Radial regressions
The IVW Radial regression [18] is a modified IVW regression of the form of
beta-wald = Beta_radial*(sqrt(weight)) + error
Where beta-wald refers to the wald-type ratio estimator, and the weight represents the original user defined inverse-variance weights. The IVW Radial allows for a more accurate identification of outliers as compared to the traditional IVW regression. The Radial MR R package is readily available for download from https://github.com/WSpiller/RadialMR.

Preparation of GWAS on sun-exposure and pigmentation phenotypes for multivariable MR analysis

We used self-reported data from the UK Biobank to perform a GWAS on hair colour, skin colour, sunburns to evaluate these traits alongside with 25(OH)D in a formal multivariable MR framework. For individuals reporting information across multiple instances (through multiple visits), only self-reported data from the first instance (instance 0) were used. A brief description of the individual GWAS for each trait examined and performed only among UKB participants of white British ancestry is provided below.

Episodes of childhood sunburn. Data for hair colour in the UK Biobank was collated from the UKB field-ID: 1737 from 498 430 participants where the question “Before the age of 15, how many times did you suffer sunburn that was painful for at least 2 days or caused blistering?” was asked. The mean of the reported episodes of childhood sunburn was 1.627 (with sd of 5). Individuals that report more than 100 episodes were excluded. The GWAS for the facial aging trait was performed as a quantitative phenotype through a linear mixed model implemented in BOLT-LMM [20].

Facial aging. Data for hair colour in the UK Biobank was collated from the UKB field-ID: 1757 based on 498 740 participants where the question “Do people say that you look older/younger/about your age?” was asked. We re-coded the phenotype to compare individuals that self-report to look younger (coded as 1) against those that reported otherwise (coded as 0). The GWAS for the facial aging trait was performed as a quantitative phenotype through a linear mixed model implemented in BOLT-LMM [20], however the equivalent log(OR) estimates can be algebraically derived.

Hair colour (Red Hair yes/no). Data for hair colour in the UK Biobank was collated from the UKB field-ID: 1747 based on 501,631 participants where the question “What best describes your natural hair colour? (If your hair colour is grey, the colour before you went grey)” was asked. Given previous findings on how the presence of natural red hair are associated with increased

risk of skin cancers, we re-coded the hair colour phenotype (coded as 1) to contrast red hair individuals against individuals of other hair colour (coded as 0). The GWAS for the red hair trait was performed as a quantitative phenotype through a linear mixed model implemented in BOLT-LMM [20], however the equivalent log(OR) estimates can be algebraically derived.

Skin Colour. Data for skin colour in the UK Biobank was collated from the UKB field-ID: 1717 based on 501,631 participants where the question "What best describes the colour of your skin without tanning?" was asked. We then converted the individuals' response into an ordinal scale {1: very fair; 2: fair; 3: light olive; 4: dark olive; 5: brown; 6: black}, with phenotypes from individuals that did not or refused to response set to NA.

Daily duration of walking and vigorous activity. Data for duration of vigorous activity in the UK Biobank was collated from the UKB field-ID: 914 where the question "How many minutes did you usually spend doing vigorous activities on a typical DAY?" was asked while data for duration of walks (UKB ID-field) were available for 479 987 individuals that answered the question "How many minutes did you usually spend walking on a typical DAY?". Responses on both questions were provided in units of minutes/day with an average value of 44.57 min/day (sd of 47.25) for duration of vigorous activity and 60.96 min/day (sd of 77.03) for walking duration. The raw values for both duration of vigorous activity and walking were retained without transformation. These GWASs were performed as quantitative phenotypes through a linear mixed model implemented in BOLT-LMM [20].

Selection of trait for the MVMR analysis

Genetic instruments for each trait were derived from variants with genome-wide significant associations on their respective traits. Instruments from each individual candidate trait were then merged into a single set of SNP instruments (nSNPs=333), and we extracted the association estimates between these SNPs on each of the risk factors. Prior to any MVMR analysis, we first evaluated the strength of the combined instrument in predicting each risk factor via the conditional F-statistics implemented through the strength_mvmr() function in the MVMR R package by Sanderson et al. [19] (https://github.com/WSpiller/MVMR). Traits without a conditional F-statistics > 10 were dropped, resulting in less than 4 traits for the MVMR analysis. We then recompiled the combined instrument based on the remaining 3 traits (skin colour, episodes of childhood sunburn and vitamin D) and re-evaluated the strength of the newly compiled instruments (nSNPs = 209). The conditional F-statistics for the 3 traits were 13.15 for childhood sunburn, 11.44 for skin colour and 59.40 for 25(OH)D, fulfilling the criteria for strong instrument in the MVMR setting. The subsequent multivariable MR regression was performed via the mv_multiple function available in the TwoSampleMR package. We only present the MVMR finding from the 3-trait model as a main finding in the manuscript; however, the MVMR estimate from the original 8 candidate traits can be found in Supplementary Table 10 (caution: the 8-trait MVMR estimates are vulnerable to weak instrument bias) for the reader's interest.

Supplementary Tables

Supplementary Table 1. Comparison of the proportion of 25(OH)D variance explained by SNP instruments estimated using the UKB cohort

# of variants	Instruments	Source	Cumulative r2	Largest P-value
5	Top 5 25(OH)D associated variants from Jiang et al.	SUNLIGHT	2.40%	3.08e-107
6	Top 6 25(OH)D variants from the UKBB including the rare CYP2R1 variant	SUNLIGHT, Manousaki et al. (2017)	3.00%	1.30E-35
78	All the above including novel loci	UKBB 25(OH)D GWAS	3.90%	5.00E-08

P-values were derived from (one-sided) chi squared statistics of the 25(OH)D GWAS association and unadjusted for multiple comparison.

Supplementary Table 2. Estimation of power for Mendelian randomization analyses for various cancers based on total sample size and proportion of phenotypic variance explained by 25(OH)D SNP instruments

Cancers	N	Proportion of cases	Proportion of 25(OH)D variance explained by instruments	Power to detect true OR per 1 SD increase in 25(OH)D		
				OR > 1.1	OR > 1.2	OR > 1.4
Barret's Eso. and Esophageal cancer	27438	0.37	0.045	0.37	0.89	1.00
Barret's Esophagus	23326	0.26	0.045	0.28	0.77	1.00
Esophageal carcinoma	21271	0.19	0.045	0.22	0.65	1.00
Breast cancer	228951	0.54	0.045	0.99	1.00	1.00
ER+ breast cancer	175475	0.40	0.045	0.99	1.00	1.00
ER- breast cancer	127442	0.17	0.045	0.80	1.00	1.00
Endometrial cancer	121885	0.11	0.045	0.63	1.00	1.00
EC Endometrial cancer	54884	0.16	0.045	0.43	0.94	1.00
NEEC Endometrial cancer	36677	0.03	0.045	0.11	0.28	0.78
Lung Cancer	27209	0.42	0.045	0.38	0.89	1.00
Lung adenocarcinoma	18336	0.19	0.045	0.20	0.59	0.99
Squamous cell lung cancer	18313	0.18	0.045	0.19	0.57	0.99
Melanoma skin cancer	42399	0.38	0.045	0.53	0.98	1.00
SCC (Keratinocyte cancers)	292755	0.03	0.045	0.50	0.97	1.00
BCC (Keratinocyte cancers)	293989	0.05	0.045	0.70	1.00	1.00
Neuroblastoma	4881	0.33	0.045	0.10	0.26	0.70
Ovarian Cancer	66450	0.38	0.045	0.73	1.00	1.00
ClearCell	42307	0.03	0.045	0.12	0.32	0.84
Endometrioid	43751	0.06	0.045	0.18	0.55	0.99
HighGrade serous	53978	0.24	0.045	0.54	0.98	1.00
LowGrade serous	41953	0.02	0.045	0.09	0.23	0.68
Mucinous	42358	0.03	0.045	0.12	0.32	0.84
Pancreatic Cancer	3835	0.49	0.045	0.10	0.22	0.61
Prostate Cancer	140254	0.56	0.045	0.96	1.00	1.00

Supplementary Table 3. MR association between 25(OH)D and skin cancers excluding 25(OH)D variants* associated with skin colour, facial aging and episodes of childhood sunburn

Outcome	Method	SNPs	Pvalue	OR (95% CI)
Melanoma	MR Egger	54	0.58	1.06 (0.87 to 1.29)
Melanoma	Weighted median	54	0.18	1.14 (0.94 to 1.39)
Melanoma	Inverse variance weighted	54	0.59	1.04 (0.89 to 1.22)
Melanoma	Simple mode	54	0.61	1.16 (0.66 to 2.04)
Melanoma	Weighted mode	54	0.45	1.06 (0.91 to 1.24)
BCC	MR Egger	60	0.45	1.06 (0.91 to 1.23)
BCC	Weighted median	60	0.08	1.12 (0.98 to 1.27)
BCC	Inverse variance weighted	60	0.03	1.14 (1.01 to 1.29)
BCC	Simple mode	60	0.10	1.40 (0.95 to 2.06)
BCC	Weighted mode	60	0.06	1.11 (1.00 to 1.24)
SCC	MR Egger	60	0.21	0.88 (0.73 to 1.07)
SCC	Weighted median	60	0.90	1.01 (0.84 to 1.22)
SCC	Inverse variance weighted	60	0.93	0.99 (0.85 to 1.16)
SCC	Simple mode	60	0.42	1.25 (0.73 to 2.16)
SCC	Weighted mode	60	0.65	0.96 (0.82 to 1.13)

*the SNP rs2270318 located in the HAL gene region were also removed. SNPs excluded due to pleiotropic association with sun expsoure, time spent outdoors or pigmentation related traits include: rs10887718,rs10985827,rs11127048,rs11542462,rs11846838, rs12123821,rs17765311,rs1972994,rs2270318,rs2975734,rs3750296,rs3768013,rs4390955,rs61816761,rs61891388,rs75360998, rs7559329,rs77924615,rs7801804,rs804281,rs9536961. P-values are two-sided, derived from z-scores of the association estimate, and unadjusted for multiple comparison unless otherwise stated.

Supplementary Table 4. Reverse MR findings for changes in 25(OH)D concentration per genetically predicted doubling of odds on cancer risk.

Major Cancers	SNPs	IVW_P-value	IVW_beta	IVW_L95	IVW_U95	correction	MR-PRESSO_P-value	MR-PRESSO_beta	MR-PRESSO_L95	MR_PRESSO_U95
BEEA	10	0.74	-0.001	-0.010	0.007	N	0.74	-0.001	-0.010	0.007
Breast cancer	201	0.39	-0.002	-0.008	0.003	Y	0.68	-0.001	-0.006	0.004
Endometrial cancer	18	0.99	0.000	-0.009	0.009	N	0.99	0.000	-0.009	0.009
Melanoma skin cancer^	18	0.51	-0.003	-0.013	0.006	Y	0.26	-0.003	-0.008	0.002
SCC	14	0.43	0.004	-0.006	0.013	Y	0.18	-0.005	-0.013	0.002
BCC	46	0.67	0.002	-0.006	0.010	Y	0.13	-0.004	-0.010	0.001
Ovarian Cancer	26	0.41	-0.005	-0.017	0.007	Y	0.41	-0.004	-0.013	0.005
Prostate Cancer	84	0.13	-0.003	-0.007	0.001	Y	0.21	-0.003	-0.007	0.001

MR-PRESSO P-values are two-sided, derived from z-scores of the association estimate, and unadjusted for multiple comparison unless otherwise stated.

Supplementary Table 5. MR association between per SD increase in 25(OH)D and cancer risk using sex-specific 25(OH)D genetic instruments.

Cancer risk	Instrument set	SNPs used	Pvalue	OR (95% CI)
Breast	25(OH)D instruments in female only GWAS	33	0.50	1.02 (0.96 to 1.10)
Endometrial	25(OH)D instruments in female only GWAS	33	0.48	0.95 (0.83 to 1.09)
Ovarian	25(OH)D instruments in female only GWAS	33	0.11	0.92 (0.84 to 1.02)
Prostate	25(OH)D instruments in male only GWAS	34	0.16	1.07 (0.97 to 1.17)

P-values are two-sided, derived from z-scores of the association estimate, and unadjusted for multiple comparison unless otherwise stated.

Supplementary Table 6. Comparison of MR association between one SD increase in 25(OH)D concentration and risk of cancer(s) with/without adjustment for vitamin D supplementation use.

		NOT Adjusted for supplementation use<br><br>**Using original UKB 25(OH)D estimate from main analysis**			***Adjusted for supplementation use***<br><br>**Using Revez et al. UKB 25(OH)D estimate (SNPs clumped with r2=0.001)**		
**Cancer outcome**	**Group**	**nsnps**	**P**	**OR (95% CI)**	**nsnps**	**P**	**OR (95% CI)**
Barrett's Esophagus		76	0.97	1.00 (0.84 to 1.18)	81	0.92	1.01 (0.84 to 1.22)
Esophageal carcinoma		76	0.76	0.97 (0.78 to 1.20)	81	0.59	0.94 (0.75 to 1.18)
BE and EA combined	Esophagus	76	0.82	0.98 (0.85 to 1.14)	81	0.88	0.99 (0.84 to 1.16)
All breast cancer		74	0.60	1.02 (0.94 to 1.10)	74	0.91	1.00 (0.93 to 1.08)
ER- breast cancer		74	0.94	1.00 (0.90 to 1.12)	74	0.95	1.00 (0.89 to 1.11)
ER+ breast cancer	Breast	74	0.51	1.03 (0.94 to 1.13)	74	0.81	1.01 (0.93 to 1.10)
All Endometrial cancer		75	0.32	0.93 (0.80 to 1.07)	77	0.41	0.94 (0.81 to 1.09)
EEC Endometrial cancer		75	0.32	0.92 (0.79 to 1.08)	77	0.27	0.91 (0.78 to 1.07)
NEEC Endometrial cancer	Endometrial	74	0.94	1.01 (0.73 to 1.41)	77	0.94	0.99 (0.71 to 1.38)
All EOC		76	0.03	0.90 (0.82 to 0.99)	79	0.09	0.91 (0.82 to 1.01)
ClearCell EOC		76	0.36	0.86 (0.64 to 1.18)	79	0.33	0.85 (0.62 to 1.17)
endometrioid EOC		76	0.56	0.94 (0.76 to 1.16)	79	0.56	0.93 (0.73 to 1.19)
High grade serous EOC		76	0.15	0.92 (0.82 to 1.03)	79	0.33	0.94 (0.82 to 1.07)
Low grade serous EOC		76	0.99	1.00 (0.71 to 1.40)	79	0.72	0.94 (0.66 to 1.34)
mucinous EOC	Ovarian	76	0.58	0.94 (0.74 to 1.18)	79	0.99	1.00 (0.80 to 1.26)
Prostate cancer	Prostate	75	0.46	1.07 (0.89 to 1.29)	73	0.16	1.07 (0.97 to 1.18)
Melanoma		69	0.31	1.09 (0.92 to 1.28)	74	0.17	1.13 (0.95 to 1.34)
BCC		77	0.01	1.18 (1.05 to 1.33)	81	5.97E-04	1.23 (1.09 to 1.38)
SCC	Skin	77	0.77	1.02 (0.88 to 1.19)	81	0.37	1.07 (0.92 to 1.24)

BE=Barret’s esophagus, EA=Esophageal adenocarcinoma, SCC=Squamous cell carcinoma, BCC=Basal cell carcinoma, EOC= Epithelial ovarian cancer, NEEC=Non-endometrioid endometrial cancer, EEC= Endometrioid endometrial cancer. P refers to the P-value of the MR association. P-values are two-sided, derived from z-scores of the association estimate, and unadjusted for multiple comparison unless otherwise stated. Note that we omitted cancer traits extracted from the MR-Base platform (lung cancers, neuroblastoma) for this comparison, due to the low SNP coverage in those datasets for robust evaluations. The GWAS summary data for Revez et al. 2020 (https://www.nature.com/articles/s41467-020-15421-7) can be downloaded from here: https://cnsgenomics.com/content/data

Supplementary Table 7. MR association between 25(OH)D and cancer risk under natural log(25(OH)D) and rank-transformed 25(OH)D scales.

**Outcome**	**IVW MR Estimates per unit log(25OHD) increase**		**IVW MR Estimates per SD increase in 25(OH)D (approx ~20 nmol/L increase)**	
Cancers	OR	P-value	OR	P-value
Barrett's Eso. and Esophageal cancer	0.95 (0.68 to 1.32)	0.75	0.98 (0.85 to 1.14)	0.82
Barrett's Esophagus	0.98 (0.66 to 1.46)	0.93	1.00 (0.84 to 1.18)	0.97
Esophageal adenocarcinoma	0.9 (0.56 to 1.46)	0.68	0.97 (0.78 to 1.20)	0.76
Breast cancer	1.05 (0.88 to 1.25)	0.60	1.02 (0.94 to 1.1)	0.60
ER+ breast cancer	1.07 (0.88 to 1.32)	0.49	1.03 (0.94 to 1.13)	0.51
ER- breast cancer	1.02 (0.79 to 1.3)	0.90	1.00 (0.9 to 1.12)	0.94
Endometrial cancer	0.85 (0.6 to 1.2)	0.34	0.93 (0.80 to 1.07)	0.32
EC Endometrial cancer	0.83 (0.57 to 1.21)	0.33	0.92 (0.79 to 1.08)	0.32
NEEC Endometrial cancer	1.06 (0.49 to 2.28)	0.89	1.01 (0.73 to 1.41)	0.94
Lung Cancer	1.19 (0.79 to 1.79)	0.42	0.95 (0.78 to 1.15)	0.59

Lung adenocarcinoma	1.13 (0.64 to 2.00)	0.67	0.88 (0.67 to 1.16)	0.37
Squamous cell lung cancer	1.01 (0.58 to 1.76)	0.97	0.98 (0.76 to 1.26)	0.86
Types of skin cancers				
Melanoma	1.22 (0.85 to 1.77)	0.28	1.09 (0.92 to 1.28)	0.31
Non-melanoma skin cancers	1.11 (0.91 to 1.35)	0.38	-	-
SCC^	1.08 (0.76 to 1.53)	0.66	1.02 (0.88 to 1.19)	0.77
BCC^	1.46 (1.12 to 1.9)	0.01	1.18 (1.05 to 1.33)	0.01
Neuroblastoma	0.02 (0.05 to 0.79)	0.02	0.58 (0.34 to 1.01)	0.06
Epithelial ovarian cancer	0.78 (0.63 to 0.96)	0.02	0.9 (0.82 to 0.99)	0.03
ClearCell	0.67 (0.34 to 1.34)	0.26	0.86 (0.64 to 1.18)	0.36
Endometrioid	0.87 (0.54 to 1.41)	0.57	0.94 (0.76 to 1.16)	0.56
HighGrade serous	0.82 (0.63 to 1.07)	0.14	0.92 (0.82 to 1.03)	0.15
LowGrade serous	0.95 (0.44 to 2.08)	0.90	1.00 (0.71 to 1.4)	0.99
Mucinous	0.86 (0.5 to 1.5)	0.60	0.94 (0.74 to 1.18)	0.58

Pancreatic cancer	1.27 (0.27 to 6.05)	0.76	1.00 (0.54 to 1.85)	0.99
Prostate cancer	1.15 (0.75 to 1.76)	0.53	1.07 (0.89 to 1.29)	0.46

P-values are two-sided, derived from z-scores of the association estimate, and unadjusted for multiple comparison unless otherwise stated.

Supplementary Table 8. Comparison of log(25(OH)D) effect estimates for 25(OH)D instruments reported in various studies.

SNP	GENE	From original SUNLIGHT log(25(OH)D) GWAS				log(25(OH)D) estimates in Yarmolinsky et al.				log(25(OH)D) estimates from our UKBB GWAS			
		EA	NEA	BETA	P	EA	NEA	BETA	P	EA	NEA	BETA	P
rs3755967*	GC	T	C	-0.089	4.74E-343	T	C	0.089	-	T	C	-0.083	1.8E-1246
rs12785878	DHCR7	T	G	0.036	3.80E-62	T	G	0.036	-	T	G	0.049	1.4E-346
rs10741657	CYP2R1	A	G	0.031	2.05E-46	A	G	0.031	-	A	G	0.035	2.30E-259
rs17216707	CYP24A1	T	C	0.026	8.14E-23	T	C	0.026	-	T	C	0.016	4.50E-35
rs10745742	AMDH1	T	C	0.017	1.88E-14	T	C	0.017	-	T	C	0.013	2.30E-34
rs8018720*	SEC23A	C	G	-0.017	4.72E-09	C	G	0.019	-	C	G	-0.014	8.70E-28

*Note: The direction of effect for rs3755967 and rs8018720 used in the 25(OH)D MR analyses in Yarmolinsky et al. is seemingly different from those reported in the original Jiang et al SUNLIGHT GWAS and those obtained from our UKBB GWAS findings. P-values are two-sided, derived from z-scores of the 25OHD-GWAS association estimate, and unadjusted for multiple comparison unless otherwise stated.

Supplementary Table 9. Evaluation of instrument strength for the multivariable MR analysis adjusting for pigmentation related variables via the Sanderson-Windmeijer conditional F-statistic.

Traits used in the MVMR analysis	Category	Estimated *Sanderson-Windmeijer conditional F-statistic* for each instrument: Original MVMR model using all 8 traits (n=333)	Reduced model with 25(OH)D, one chronic sun exposure trait and one pigmentation trait (n=209)
number of episodes for childhood sunburn	chronic sun exposure	7.78	13.15
Daily duration of walking	time spent outdoors	1.43	-
Facial aging	chronic sun exposure	7.21	-
Red hair (yes/no)	pigmentation	7.71	-
Skin colour	pigmentation	8.65	11.44
Sleep duration	time spent outdoors	4.79	-
Daily duration of vigorous activity	time spent outdoors	1.12	-
25(OH)D	Main exposure of interest	40.86	59.40

The conditional F-statistics for each trait applied in the MVMR are estimated using the multivariable MR (MVMR) R package. Only results based on the reduced model (vitamin D, skin colour and number of episodes for childhood sunburn) were subsequently reported in the revised manuscript. The MVMR findings based on the original 8 trait model is shown in Supplementary Table 10.

Supplementary Table 10. Multivariable MR association between 25(OH)D and skin cancers adjusted for pigmentation related variables using the original 8 candidate traits

**Risk factors in multivariate MR model**	**Outcome**	**Marginal BETA**	**SE**	**P-value**	**OR (95% CI) per unit change in risk factor**
Episodes of childhood sunburn	Melanoma	0.89	0.31	3.46E-03	2.45 (1.34 to 4.46)
Daily walking duration (mins)	Melanoma	0.01	0.01	0.48	1.01 (0.99 to 1.02)
Facial aging	Melanoma	-2.08	0.53	9.82E-05	0.13 (0.04 to 0.36)
Red hair (yes/no)	Melanoma	3.87	1.36	4.44E-03	47.85 (3.33 to >99)
Skin colour	Melanoma	0.30	0.27	0.27	1.35 (0.80 to 2.28)
Sleep duration (hours)	Melanoma	0.43	0.37	0.24	1.54 (0.75 to 3.18)
Daily duration of vigorous activities (mins)	Melanoma	-0.01	0.01	0.55	0.99 (0.97 to 1.02)
**25(OH)D concentration (SD unit)**	Melanoma	0.14	0.11	0.21	1.15 (0.92 to 1.43)
Episodes of childhood sunburn	BCC	1.83	0.21	3.08E-18	6.23 (4.13 to 9.40)
Daily walking duration (mins)	BCC	0.01	0.01	0.04	1.01 (1.00 to 1.02)
Facial aging	BCC	0.97	0.34	0.01	2.63 (1.34 to 5.17)
Red hair (yes/no)	BCC	-0.14	0.73	0.85	0.87 (0.21 to 3.65)
Skin colour	BCC	-0.52	0.18	4.69E-03	0.59 (0.41 to 0.85)
Sleep duration (hours)	BCC	-0.11	0.24	0.65	0.89 (0.56 to 1.44)
Daily duration of vigorous activities (mins)	BCC	-0.01	0.01	0.54	0.99 (0.98 to 1.01)
**25(OH)D concentration (SD unit)**	BCC	0.14	0.08	0.08	1.15 (0.99 to 1.34)
Episodes of childhood sunburn	SCC	2.61	0.26	3.84E-23	13.61 (8.12 to 22.81)
Daily walking duration (mins)	SCC	0.02	0.01	0.04	1.02 (1.00 to 1.03)
Facial aging	SCC	0.89	0.43	0.04	2.44 (1.04 to 5.71)
Red hair (yes/no)	SCC	-0.16	0.92	0.86	0.86 (0.14 to 5.15)

Skin colour	SCC	-0.22	0.23	0.34	0.80 (0.51 to 1.26)
Sleep duration (hours)	SCC	-0.08	0.31	0.78	0.92 (0.50 to 1.68)
Daily duration of vigorous activities (mins)	SCC	0.00	0.01	0.68	1.00 (0.97 to 1.02)
**25(OH)D concentration (SD unit)**	SCC	0.08	0.10	0.40	1.09 (0.89 to 1.32)

BCC= Basal Cell Carcinoma; SCC= Squamous Cell Carcinoma. Marginal BETA reflects the marginal magnitude of association between the genetic effect of 25(OH)D SNPs exert on the risk factor of interest and skin cancer outcomes after conditioning on the genetic effect on remaining risk factors. Both the red hair and facial aging trait are binary phenotypes; hence the resultant marginal OR cannot be readily interpreted. Note that these estimates are highly vulnerable to weak instrument bias since some traits included in the model did not satisfy the strong instrument criteria (see Supplementary Table 8). All P-values are two-sided, derived from z-scores of the association estimate, and unadjusted for multiple comparison unless otherwise stated.

Supplementary Figures

Note: MR Scatter plots and funnel plots are not shown for cancer traits obtained from the MR-Base database. Those plots can be readily generated directly from the MR-Base platform.

Supplementary Figure 1. Scatter plot for the MR association between 25(OH)D and breast cancer risk. The upper panel represents the scatter plot for overall risk of breast cancer; lower panel represents findings for ER-negative and ER-positive breast cancers. The slope of each fitted line (highlighted in different colours) reflect the MR causal estimate derived from alternative 2SMR estimators. Each datapoint (n=74 SNPs) refers to the beta estimate of the exposure (vitaminD) and outcome(cancer risk) association, with the error bars reflecting the standard error on the estimate.

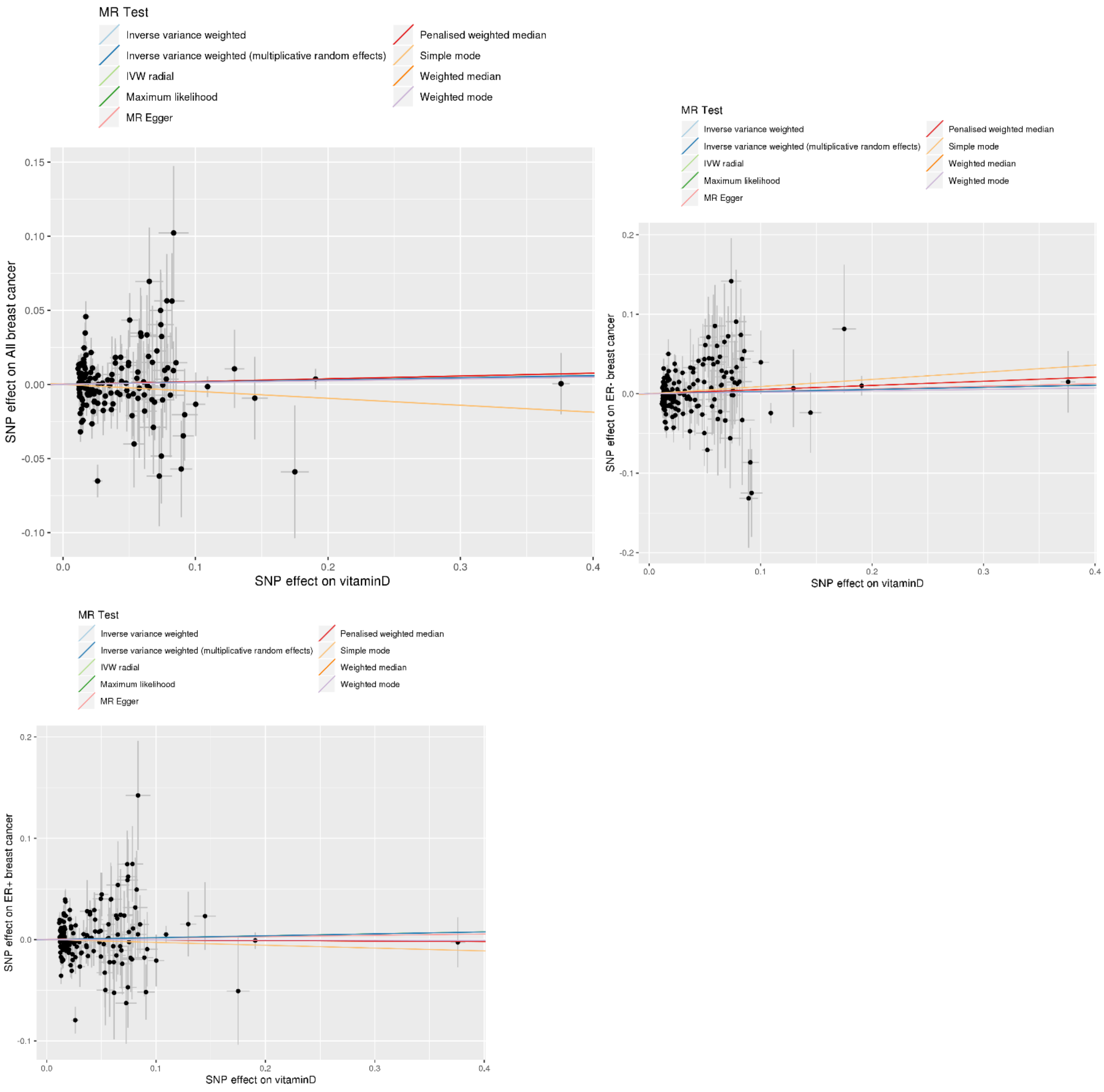

Supplementary Figure 2. Scatter plot for the MR association between 25(OH)D and endometrial cancer risk. The upper panel represents the scatter plot for overall risk of breast cancer; lower panel represents findings for Endometrioid (EEC) and Non-Endometrioid (NEEC) endometrial cancer. The slope of each fitted line (highlighted in different colours) reflect the MR causal estimate derived from alternative 2SMR estimators. Each datapoint (n=75 SNPs) refers to the beta estimate of the exposure (vitaminD) and outcome(cancer risk) association, with the error bars reflecting the standard error on the estimate.

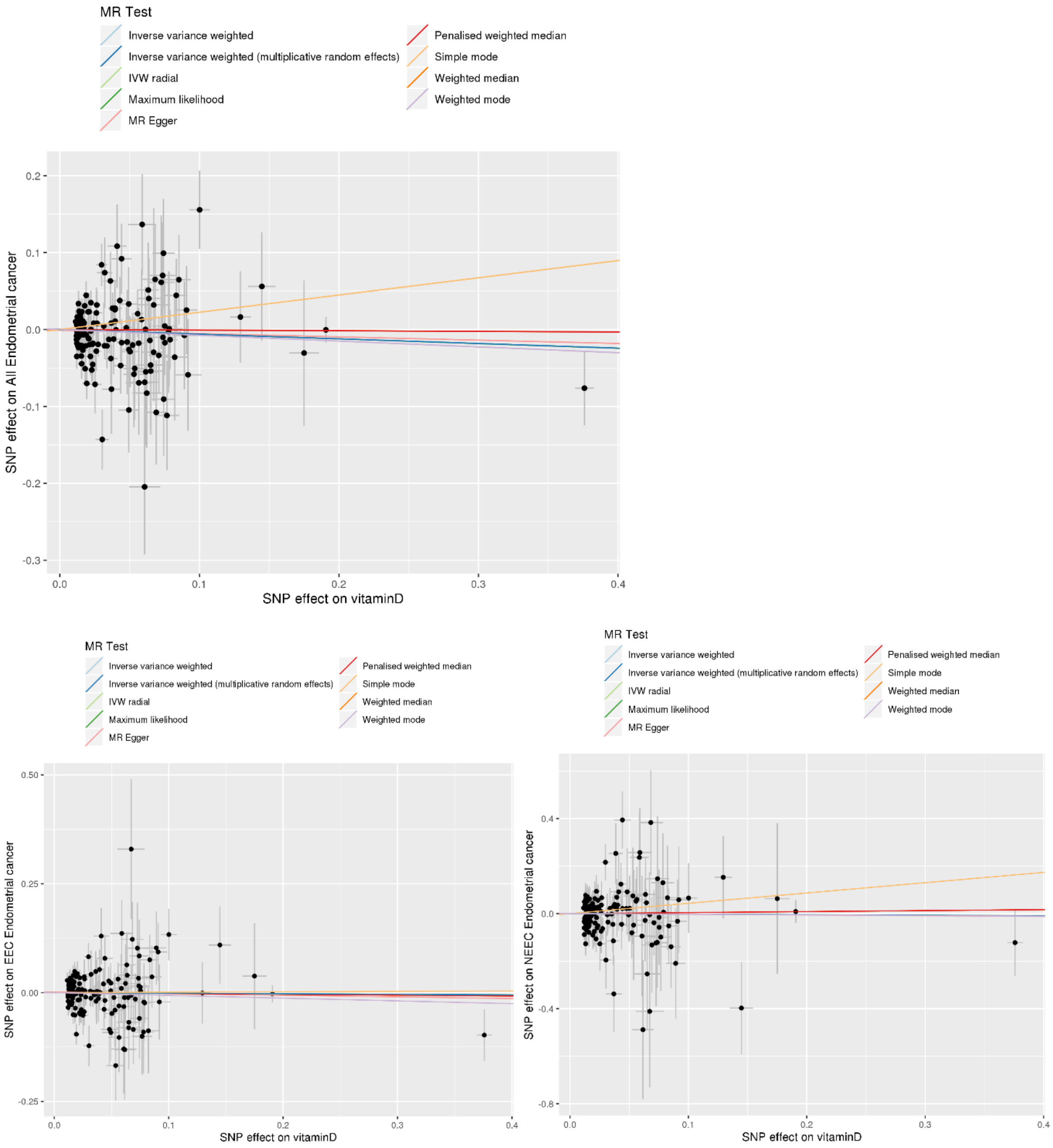

Supplementary Figure 3. Scatter plot for the MR association between 25(OH)D and EOC risk. Each panel represent the scatter plot for (A) overall risk of EOC; (B) clearcell EOC; (C) endometrioid EOC; (D) mucinous EOC; (E) high grade serous EOC and (F) low grade serous EOC. The slope of each fitted line (highlighted in different colours) reflect the MR causal estimate derived from alternative 2SMR estimators. Each datapoint (n=76 SNPs) refers to the beta estimate of the exposure (vitaminD) and outcome(cancer risk) association, with the error bars reflecting the standard error on the estimate.

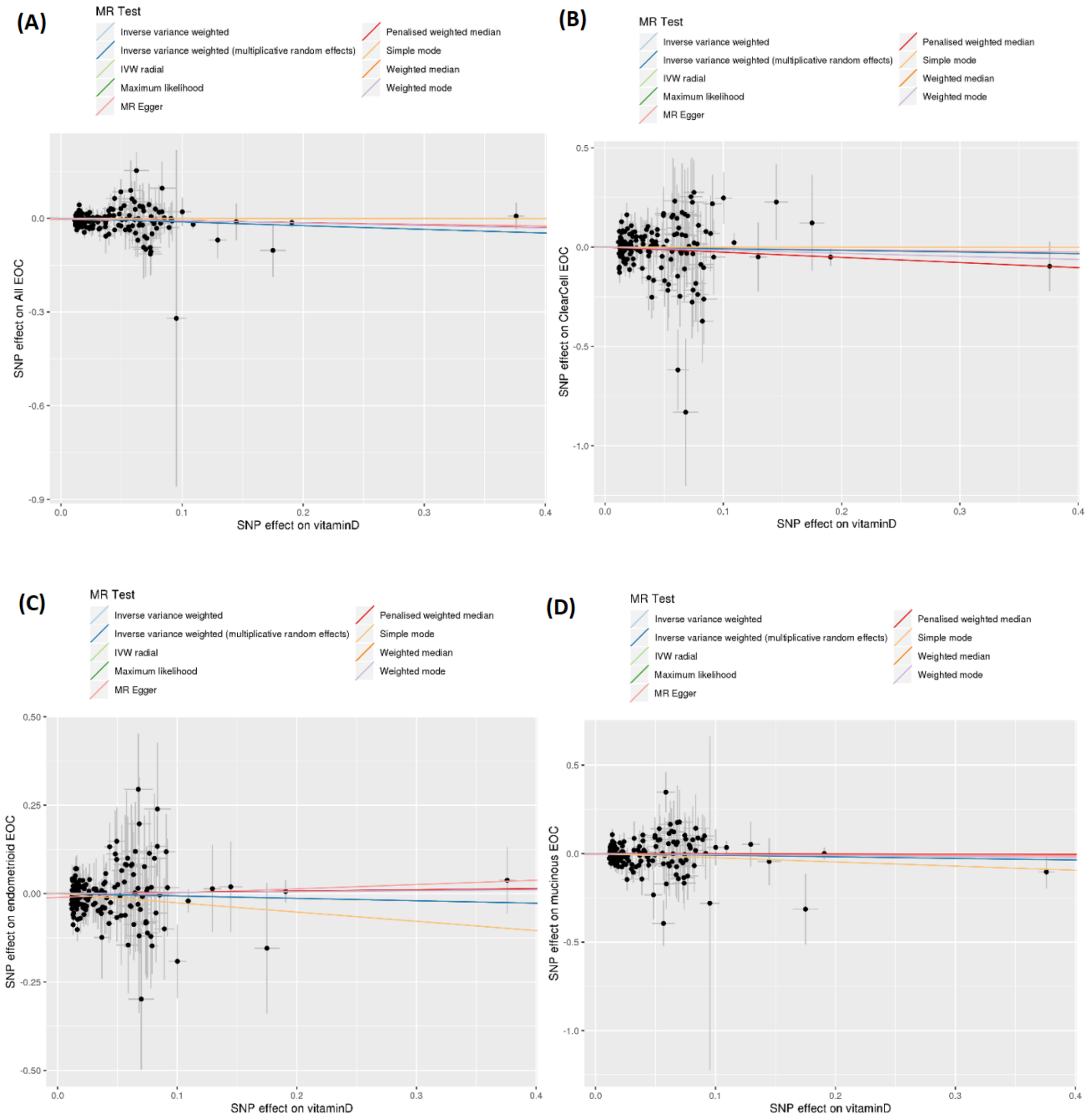

(E)

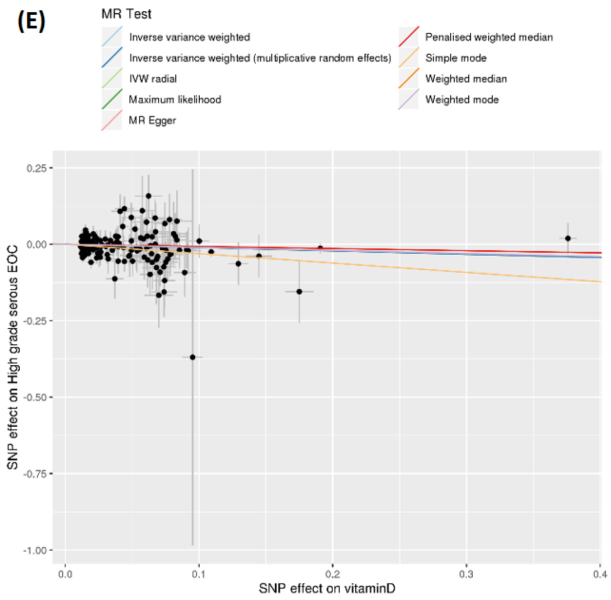
MR Test
Inverse variance weighted
Inverse variance weighted (multiplicative random effects)
IVW radial
Maximum likelihood
MR Egger
Penalised weighted median
Simple mode
Weighted median
Weighted mode
SNP effect on High grade serous EOC
SNP effect on vitaminD
0.25
0.00
-0.25
-0.50
-0.75
-1.00
0.0
0.1
0.2
0.3
0.4

(F)

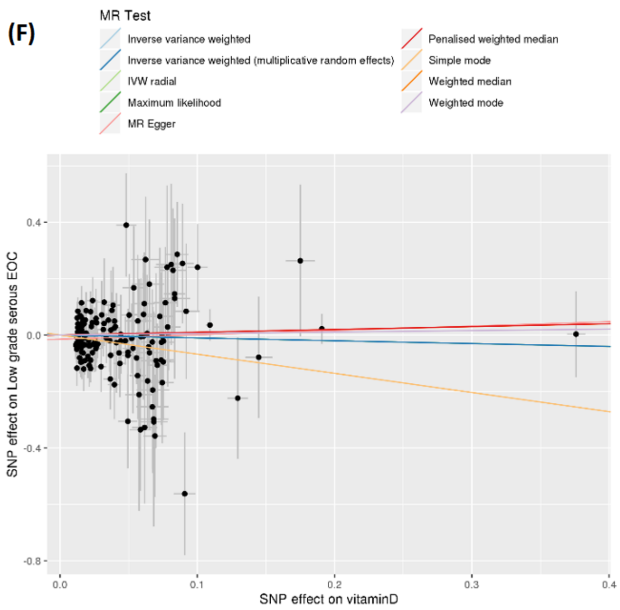
MR Test
Inverse variance weighted
Inverse variance weighted (multiplicative random effects)
IVW radial
Maximum likelihood
MR Egger
Penalised weighted median
Simple mode
Weighted median
Weighted mode
SNP effect on Low grade serous EOC
SNP effect on vitaminD
0.4
0.0
-0.4
-0.8
0.0
0.1
0.2
0.3
0.4

Supplementary Figure 4. Scatter plot for the MR association between 25(OH)D and prostate cancer risk. The slope of each fitted line (highlighted in different colours) reflect the MR causal estimate derived from alternative 2SMR estimators. Each datapoint (n=75 SNPs) refers to the beta estimate of the exposure (vitaminD) and outcome (cancer risk) association, with the error bars reflecting the standard error on the estimate.

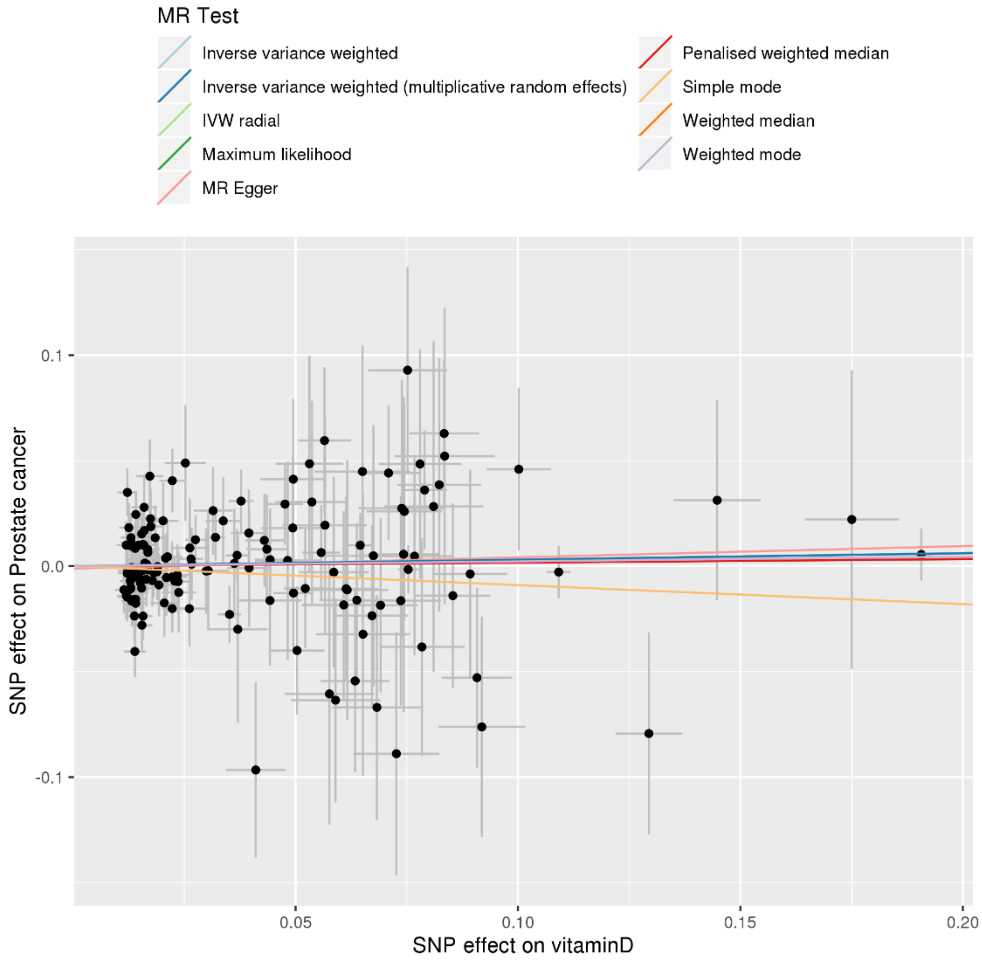

Supplementary Figure 5. Scatter plot for the MR association between 25(OH)D and the risk of BE and EA combined (BEEA). The upper panel represent the scatter plot for overall risk of BEEA; lower panel represent findings for risk of BE and EA separately. The slope of each fitted line (highlighted in different colours) reflect the MR causal estimate derived from alternative 2SMR estimators. Each datapoint (n=76 SNPs) refers to the beta estimate of the exposure (vitaminD) and outcome(cancer risk) association, with the error bars reflecting the standard error on the estimate.

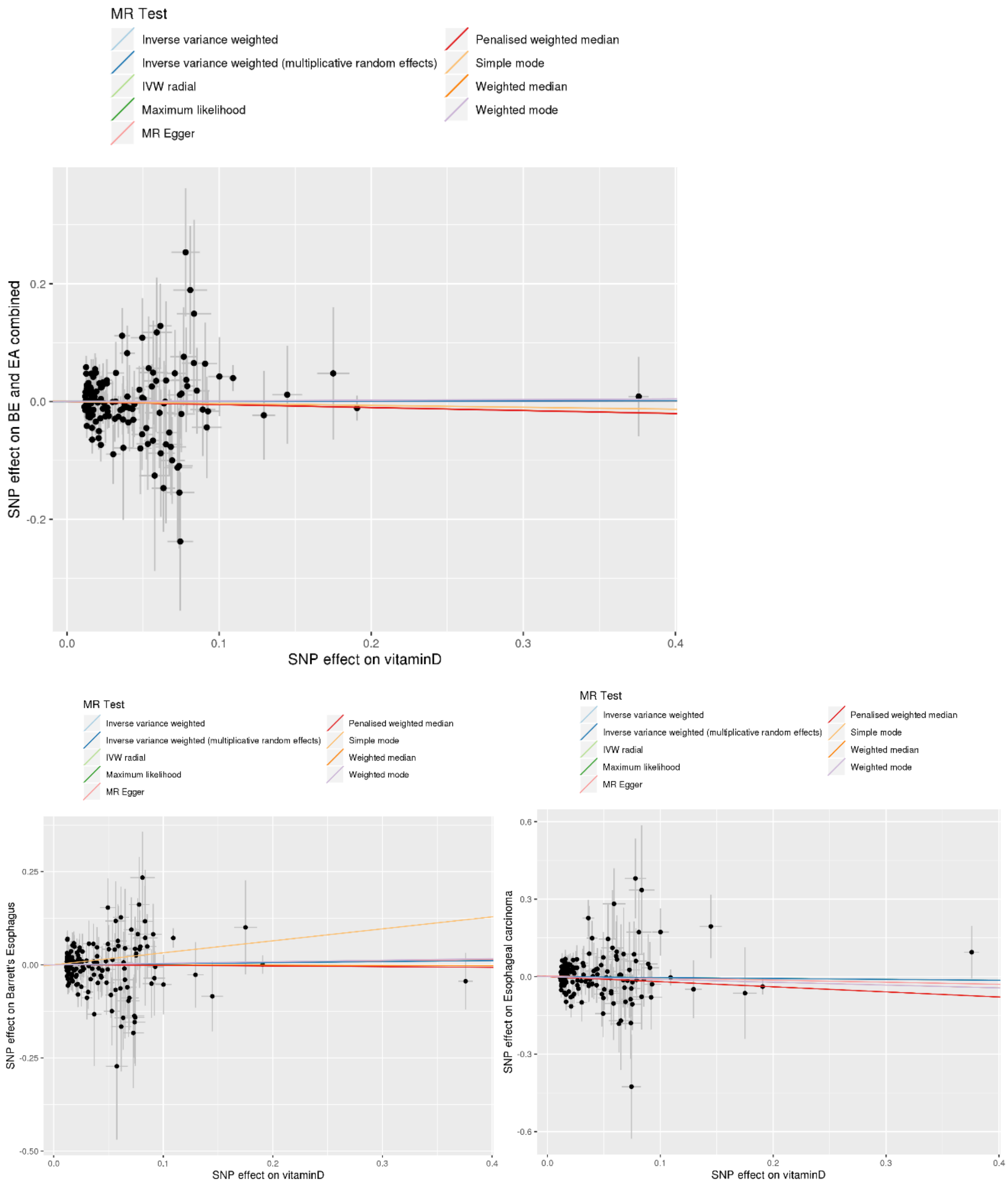

Supplementary Figure 6. Scatter plot for the MR association between 25(OH)D and risk of skin cancers. The upper panel represents the scatter plot for overall risk of melanoma; lower panel represent findings for non-melanoma skin cancers, i.e. basal cell carcinoma (BCC) and squamous cell carcinoma (SCC). The slope of each fitted line (highlighted in different colours) reflect the MR causal estimate derived from alternative 2SMR estimators. Each datapoint (n=69 SNPs for melanoma; n=77 SNPs for BCC and SCC) refers to the beta estimate of the exposure (vitaminD) and outcome(cancer risk) association, with the error bars reflecting the standard error on the estimate.

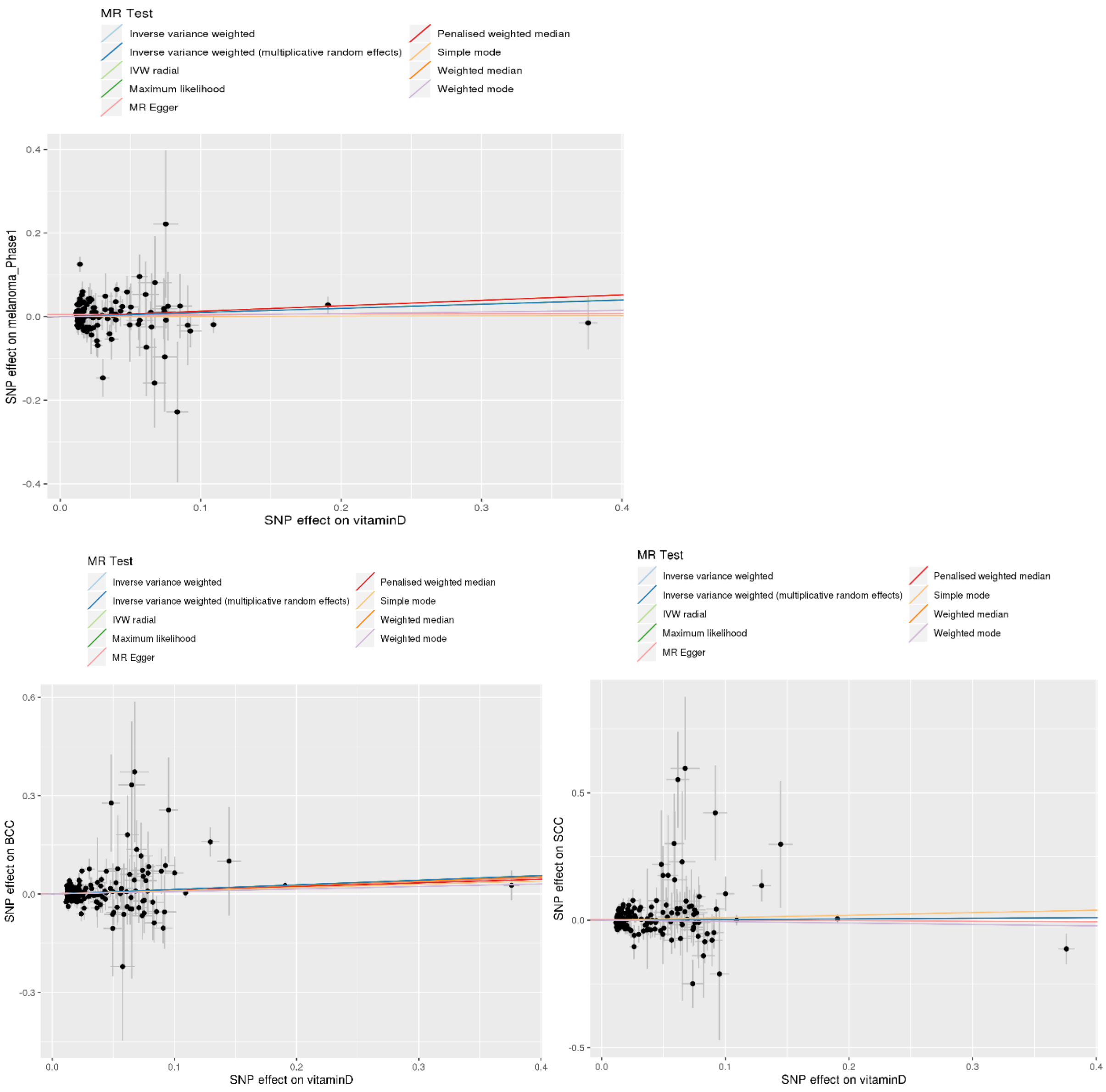

Supplementary Figure 7. Funnel plots for the MR associations between 25(OH)D and cancer risk. Funnel plots for individual subtype analyses were not shown. Most funnel plots show strong pattern of symmetry, the lack of datapoints on the left/right upper area of the distribution indicate minimal bias due to outlier effects. Panel (A) Breast cancer risk (B) Ovarian cancer risk (C) Endometrial cancer risk (D) Prostate cancer risk (E) Risk of Barret's esophagus and Esophageal cancer combined (F) Risk of melanoma (G) Risk of BCC (H) Risk of SCC.

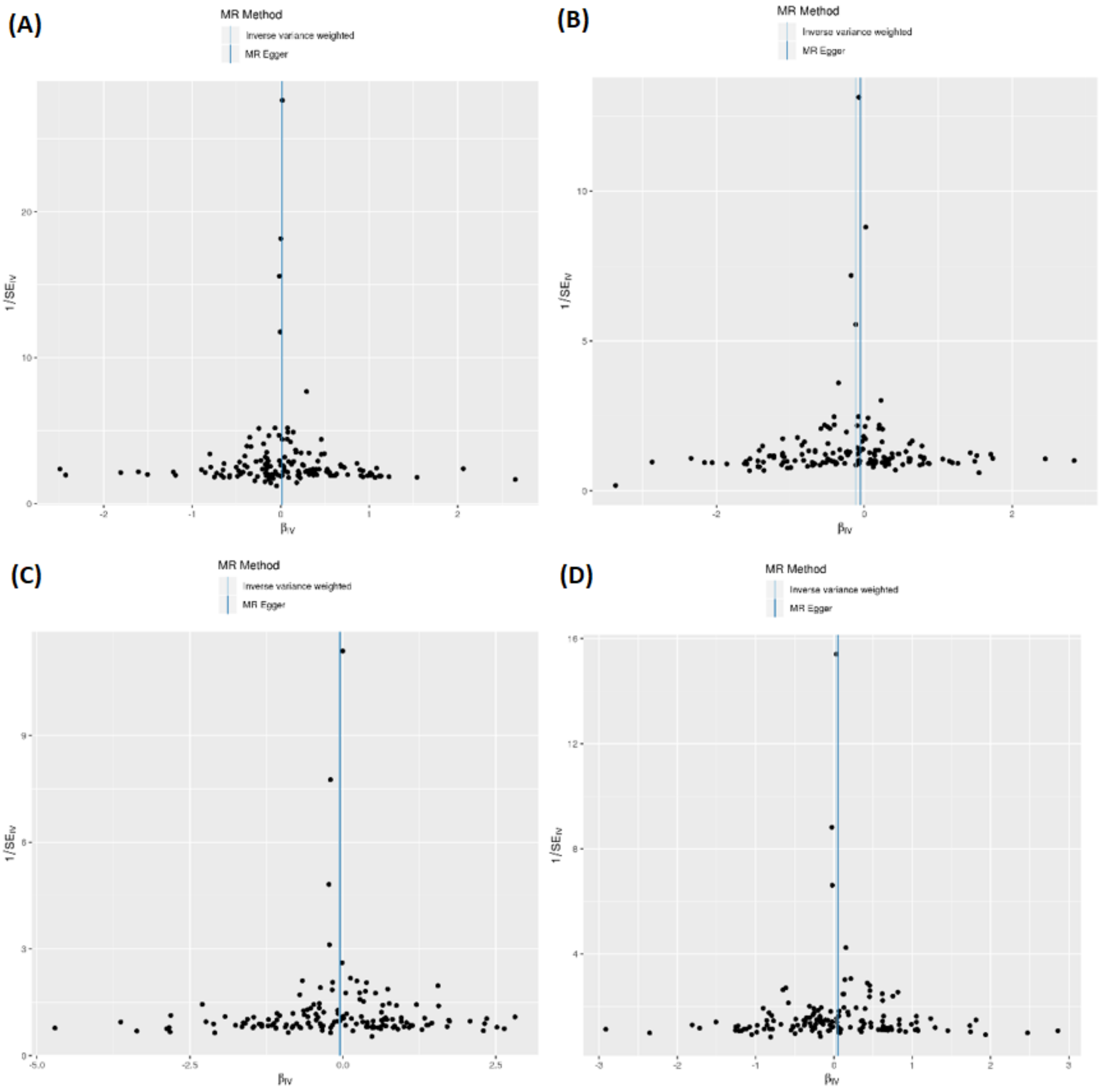

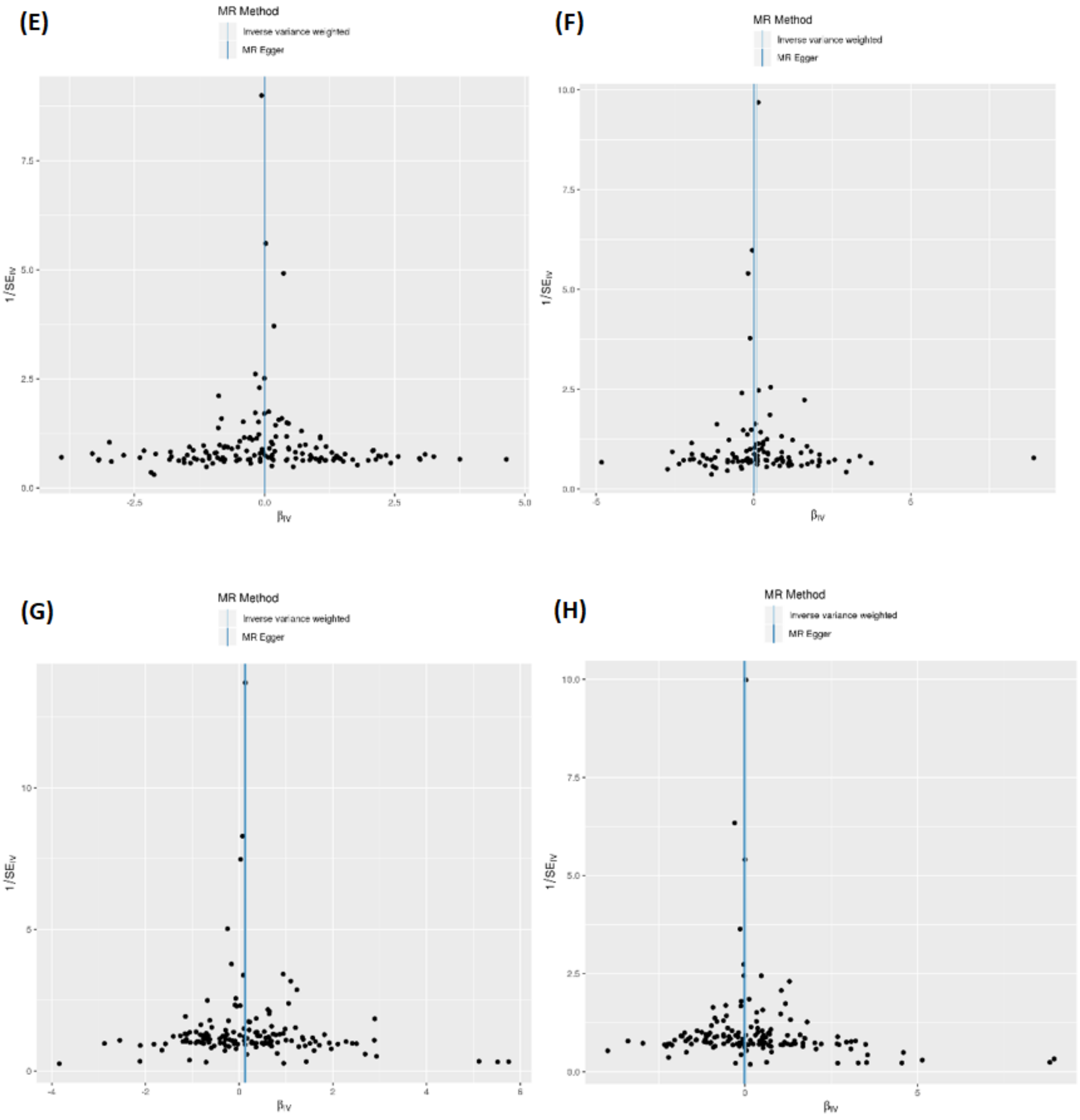
(E)
MR Method
Inverse variance weighted
MR Egger
1/SE_IV
β_IV
(F)
MR Method
Inverse variance weighted
MR Egger
1/SE_IV
β_IV
(G)
MR Method
Inverse variance weighted
MR Egger
1/SE_IV
β_IV
(H)
MR Method
Inverse variance weighted
MR Egger
1/SE_IV
β_IV

Supplementary Figure 8. Sketch diagram illustrating potential inflation of MR OR due to ascertainment bias on history of skin disorder for the MR findings on vitamin D and skin cancer risk.

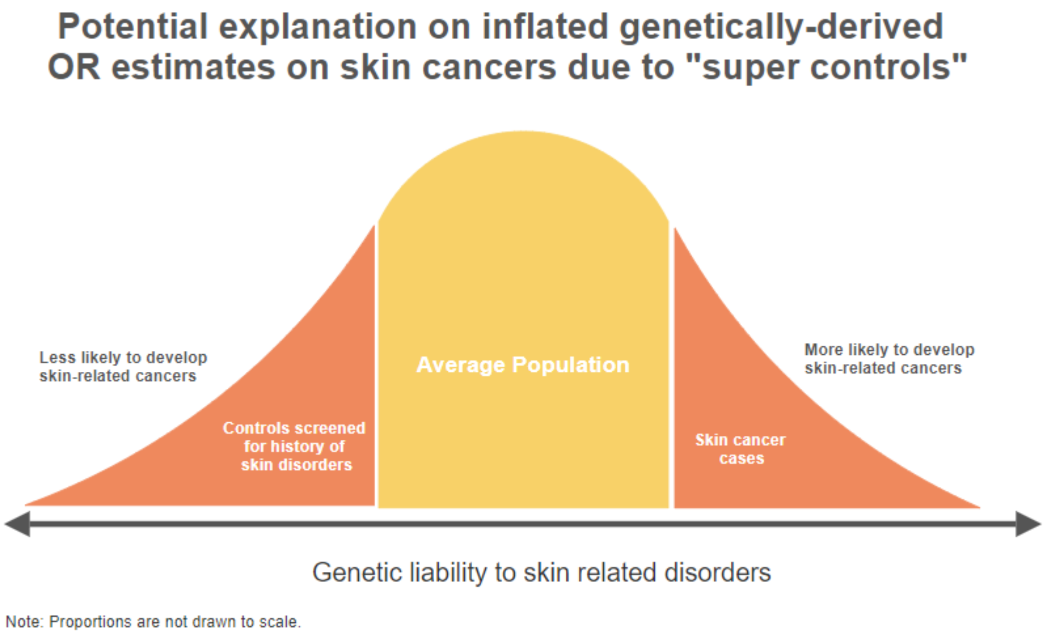

References

1. Brion M-JA, Shakhbazov K, Visscher PM. Calculating statistical power in Mendelian randomization studies. *Int J Epidemiol*. 2013 Oct;**42**(5):1497–1501.

2. Bulik-Sullivan BK, Loh P-R, Finucane HK, et al. LD Score regression distinguishes confounding from polygenicity in genome-wide association studies. *Nat Genet*. 2015 Mar;**47**(3):291–295.

3. Olsen CM, Green AC, Neale RE, et al. Cohort profile: the QSkin Sun and Health Study. *Int J Epidemiol*. 2012 Aug;**41**(4):929–929i.

4. Stoeklé H-C, Mamzer-Bruneel M-F, Vogt G, Hervé C. 23andMe: a new two-sided data-banking market model. *BMC Med Ethics*. 2016 Mar 31;**17**:19.

5. Liyanage UE, Law MH, Han X, et al. Combined analysis of keratinocyte cancers identifies novel genome-wide loci. *Hum Mol Genet*. 2019 Sep 15;**28**(18):3148–3160.

6. Chahal HS, Lin Y, Ransohoff KJ, et al. Genome-wide association study identifies novel susceptibility loci for cutaneous squamous cell carcinoma. *Nat Commun*. 2016 Jul 18;**7**:12048.

7. Chahal HS, Wu W, Ransohoff KJ, et al. Genome-wide association study identifies 14 novel risk alleles associated with basal cell carcinoma. *Nat Commun*. 2016 Aug 19;**7**:12510.

8. Michailidou K, Lindström S, Dennis J, et al. Association analysis identifies 65 new breast cancer risk loci. *Nature*. 2017 Nov 2;**551**(7678):92–94.

9. Schumacher FR, Al Olama AA, Berndt SI, et al. Association analyses of more than 140,000 men identify 63 new prostate cancer susceptibility loci. *Nat Genet*. 2018 Jul;**50**(7):928–936.

10. Phelan CM, Kuchenbaecker KB, Tyrer JP, et al. Identification of 12 new susceptibility loci for different histotypes of epithelial ovarian cancer. *Nat Genet*. 2017 May;**49**(5):680–691.

11. Wang Y, McKay JD, Rafnar T, et al. Rare variants of large effect in BRCA2 and CHEK2 affect risk of lung cancer. *Nat Genet*. 2014 Jul;**46**(7):736–741.

12. Law MH, Bishop DT, Lee JE, et al. Genome-wide meta-analysis identifies five new susceptibility loci for cutaneous malignant melanoma. *Nat Genet*. 2015 Sep;**47**(9):987–995.

13. Gharahkhani P, Fitzgerald RC, Vaughan TL, et al. Genome-wide association studies in oesophageal adenocarcinoma and Barrett's oesophagus: a large-scale meta-analysis. *Lancet Oncol*. 2016 Oct;**17**(10):1363–1373.

14. Bowden J, Davey Smith G, Haycock PC, Burgess S. Consistent Estimation in Mendelian Randomization with Some Invalid Instruments Using a Weighted Median Estimator. *Genet Epidemiol*. 2016 May;**40**(4):304–314.

15. Hartwig FP, Davey Smith G, Bowden J. Robust inference in summary data Mendelian randomization via the zero modal pleiotropy assumption. *Int J Epidemiol*. 2017 Dec 1;**46**(6):1985–1998.

16. Burgess S, Thompson SG. Interpreting findings from Mendelian randomization using the MR-Egger method. *Eur J Epidemiol*. 2017 May;**32**(5):377–389.

17. Verbanck M, Chen C-Y, Neale B, Do R. Detection of widespread horizontal pleiotropy in causal relationships inferred from Mendelian randomization between complex traits and diseases. *Nat Genet*. 2018 May;**50**(5):693–698.

18. Bowden J, Spiller W, Del Greco M F, et al. Improving the visualization, interpretation and analysis of two-sample summary data Mendelian randomization via the Radial plot and Radial regression. *Int J Epidemiol*. 2018 Aug 1;**47**(4):1264–1278.

19. Sanderson E, Davey Smith G, Windmeijer F, Bowden J. An examination of multivariable Mendelian randomization in the single-sample and two-sample summary data settings. *Int J Epidemiol*. 2019 Jun 1;**48**(3):713–727.

20. Loh P-R, Kichaev G, Gazal S, Schoech AP, Price AL. Mixed-model association for biobank-scale datasets. *Nat Genet*. 2018 Jul;**50**(7):906–908.

Supplementary Note

for manuscript **A comprehensive re-assessment of the association between vitamin D and cancer susceptibility using Mendelian randomization**

Detailed Acknowledgement

The PRACTICAL consortium

The Prostate cancer genome-wide association analyses are supported by the Canadian Institutes of Health Research, European Commission’s Seventh Framework Programme grant agreement n° 223175 (HEALTH-F2-2009-223175), Cancer Research UK Grants C5047/A7357, C1287/A10118, C1287/A16563, C5047/A3354, C5047/A10692, C16913/A6135, and The National Institute of Health (NIH) Cancer Post-Cancer GWAS initiative grant: No. 1 U19 CA 148537-01 (the GAME-ON initiative).

We would also like to thank the following for funding support: The Institute of Cancer Research and The Everyman Campaign, The Prostate Cancer Research Foundation, Prostate Research Campaign UK (now PCUK), The Orchid Cancer Appeal, Rosetrees Trust, The National Cancer Research Network UK, The National Cancer Research Institute (NCRI) UK. We are grateful for support of NIHR funding to the NIHR Biomedical Research Centre at The Institute of Cancer Research and The Royal Marsden NHS Foundation Trust.

The Prostate Cancer Program of Cancer Council Victoria also acknowledge grant support from The National Health and Medical Research Council, Australia (126402, 209057, 251533, , 396414, 450104, 504700, 504702, 504715, 623204, 940394, 614296,), VicHealth, Cancer Council Victoria, The Prostate Cancer Foundation of Australia, The Whitten Foundation, PricewaterhouseCoopers, and Tattersall’s. EAO, DMK, and EMK acknowledge the Intramural Program of the National Human Genome Research Institute for their support. Genotyping of the OncoArray was funded by the US National Institutes of Health (NIH) [U19 CA 148537 for ELucidating Loci Involved in Prostate cancer SuscEptibility (ELLIPSE) project and X01HG007492 to the Center for Inherited Disease Research (CIDR) under contract number HHSN268201200008I] and by Cancer Research UK grant A8197/A16565. Additional analytic support was provided by NIH NCI U01 CA188392 (PI: Schumacher).

Funding for the iCOGS infrastructure came from: the European Community’s Seventh Framework Programme under grant agreement n° 223175 (HEALTH-F2-2009-223175) (COGS), Cancer Research UK (C1287/A10118, C1287/A 10710, C12292/A11174, C1281/A12014, C5047/A8384, C5047/A15007, C5047/A10692, C8197/A16565), the National Institutes of Health (CA128978) and Post-Cancer GWAS initiative (1U19 CA148537, 1U19 CA148065 and 1U19 CA148112 – the GAME-ON initiative), the Department of Defence (W81XWH-10-1-0341), the Canadian Institutes of Health Research (CIHR) for the CIHR Team in Familial Risks of Breast Cancer, Komen Foundation for the Cure, the Breast Cancer Research Foundation, and the Ovarian Cancer Research Fund. The BPC3 was supported by the U.S. National Institutes of Health, National Cancer Institute (cooperative agreements U01-

CA98233 to D.J.H., U01-CA98710 to S.M.G., U01-CA98216 toE.R., and U01-CA98758 to B.E.H., and Intramural Research Program of NIH/National Cancer Institute, Division of Cancer Epidemiology and Genetics). CAPS GWAS study was supported by the Swedish Cancer Foundation (grant no 09-0677, 11-484, 12-823), the Cancer Risk Prediction Center (CRisP; www.crispcenter.org), a Linneus Centre (Contract ID 70867902) financed by the Swedish Research Council, Swedish Research Council (grant no K2010-70X-20430-04-3, 2014-2269) PEGASUS was supported by the Intramural Research Program, Division of Cancer Epidemiology and Genetics, National Cancer Institute, National Institutes of Health.

The BCAC consortium

The breast cancer genome-wide association analyses were supported by the Government of Canada through Genome Canada and the Canadian Institutes of Health Research, the 'Ministère de l'Économie, de la Science et de l'Innovation du Québec' through Genome Québec and grant PSR-SIIRI-701, The National Institutes of Health (U19 CA148065, X01HG007492), Cancer Research UK (C1287/A10118, C1287/A16563, C1287/A10710) and The European Union (HEALTH-F2-2009-223175 and H2020 633784 and 634935). All studies and funders are listed in Michailidou et al (2017).

The OCAC consortium

The Ovarian Cancer Association Consortium (OCAC) is supported by a grant from the Ovarian Cancer Research Fund thanks to donations by the family and friends of Kathryn Sladek Smith (PPD/RPCI.07). The scientific development and funding for this project were in part supported by the US National Cancer Institute GAME-ON Post-GWAS Initiative (U19-CA148112). This study made use of data generated by the Wellcome Trust Case Control consortium that was funded by the Wellcome Trust under award number 076113. Results published here are in part based upon data generated by The Cancer Genome Atlas Pilot Project established by the National Cancer Institute and National Human Genome Research Institute (dbGap accession number phs000178.v8.p7). The OCAC OncoArray genotyping project was funded through grants from the U.S. National Institutes of Health (CA1X01HG007491-01 (C.I.A.), U19-CA148112 (T.A.S.), R01-CA149429 (C.M.P.) and R01-CA058598 (M.T.G.); Canadian Institutes of Health Research (MOP-86727 (L.E.K.) and the Ovarian Cancer Research Fund (A.B.). The COGS project was funded through a European Commission's Seventh Framework Programme grant (agreement number 223175 - HEALTH-F2-2009-223175). All studies and funders are listed in Phelan et al (2017).

The ECAC consortium

The iCOGS and OncoArray endometrial cancer analysis for data from the Endometrial Cancer Association Consortium (ECAC) were supported by NHMRC project grants (ID#1031333 and ID#1109286). Funding for the iCOGS infrastructure came from: the European Community's Seventh Framework Programme under grant agreement no. 223175 (HEALTH-F2-2009-223175) (COGS), Cancer Research UK (C1287/A10118, C1287/A10710, C12292/A11174, C1281/A12014, C5047/A8384, C5047/A15007, C5047/A10692, C8197/A16565), the National

Institutes of Health (CA128978) and Post-Cancer GWAS initiative (1U19 CA148537, 1U19 CA148065 and 1U19 CA148112—the GAME-ON initiative), the Department of Defence (W81XWH-10-1-0341), the Canadian Institutes of Health Research (CIHR) for the CIHR Team in Familial Risks of Breast Cancer, Komen Foundation for the Cure, the Breast Cancer Research Foundation, and the Ovarian Cancer Research Fund. Please see (O'Mara et al. 2018) for complete acknowledgements for the individual studies in ECAC.

The QSkin Genetic study

The QSkin Study is conducted by a team of researchers from the QIMR Berghofer Medical Research Institute, Brisbane, Australia. We are grateful to the National Health and Medical Research Council of Australia (NHMRC) for funding (APP1063061 and APP1185416), and to the Queenslanders who have willingly given their time to take part.

The GenoMEL consortium

The Genetics of Melanoma (GenoMEL) study (http://www.genomel.org/) was funded by the European Commission under the 6th Framework Programme (contract no. LSHC-CT-2006-018702), by Cancer Research UK Programme Awards (C588/A4994 and C588/A10589), by a Cancer Research UK Project Grant (C8216/A6129) and by another grant from the US National Institutes of Health (NIH; CA83115). This research was also supported by the intramural Research Program of the NIH, National Cancer Institute (NCI), Division of Cancer Epidemiology and Genetics. The GenoMEL GWAS study makes use of data generated by the Wellcome Trust Case Control Consortium (http://www.wtccc.org.uk/). A full list of the investigators who contributed to the generation of the data is available from their website (see URLs). Funding for the project was provided by the Wellcome Trust under award 076113. Please see (Law et al. 2015) for acknowledgements of specific studies used in the Law et al. 2015 melanoma GWAS meta-analysis applied in this study.

The Esophageal Cancer consortium (BEACON, CAMBRIDGE, OXFORD and the BONN study)

The Esophageal Cancer consortium is a collection of various studies and the Barrett's Esophagus and Esophageal Adenocarcinoma Consortium (BEACON). We thank all patients and controls for participating in the BEACON GWAS study. The MD Anderson controls were drawn from dbGaP (study accession: phs000187.v1.p1). Genotyping of these controls were done through the University of Texas MD Anderson Cancer Center (UTMDACC) and the Johns Hopkins University Center for Inherited Disease Research (CIDR). We acknowledge the principal investigators of this study: Christopher Amos, Qingyi Wei, and Jeffrey E Lee. Controls from the Genome-Wide Association Study of Parkinson Disease were obtained from dbGaP (study accession: phs000196.v2.p1). This work, in part, used data from the National Institute of Neurological Disorders and Stroke (NINDS) dbGaP database from the CIDR:NeuroGenetics Research Consortium Parkinson's disease study. We would also like to acknowledge the

principal investigators and coinvestigators of this study: Haydeh Payami, John Nutt, Cyrus Zabetian, Stewart Factor, Eric Molho, and Donald Higgins. Controls from the Chronic Renal Insufficiency Cohort (CRIC) were drawn from dbGaP (study accession: phs000524.v1.p1). The CRIC study was done by the CRIC investigators and supported by the National Institute of Diabetes and Digestive and Kidney Diseases (NIDDK). Phenotypic and genetic data and samples from CRIC reported here were supplied by NIDDK Central Repositories. We acknowledge the principal investigators and the project officer of this study: Harold I Feldman, Raymond R Townsend, Lawrence J Appel, Mahboob Rahman, Akinlolu Ojo, James P Lash, Jiang He, Alan S Go, and John W Kusek. Please see (Gharahkhani et al. 2016) for study acknowledgements and funding details for the individual contributing studies that generated the esophageal cancer summary statistics applied in this study.
