# Supplementary material for: A comprehensive re-assessment of the association between vitamin D and cancer susceptibility using Mendelian randomization
Source: Nat Commun. 2021 Jan 11;12:246. doi: 10.1038/s41467-020-20368-w (PMC7801600; doi:10.1038/s41467-020-20368-w)
Supplement: Supplementary file 3 — Description of Additional Supplementary Files [file 41467_2020_20368_MOESM3_ESM.pdf]

Description of Additional Supplementary Files

File Name: Supplementary Data 1
Description: Estimated genetic effect sizes on rank transformed 25(OH)D concentration for each of the 78 SNP instrument. Association estimates derived directly from UK Biobank white British participants using BOLT-LMM.

File Name: Supplementary Data 2
Description: Estimated genetic effect sizes on natural log transformed 25(OH)D concentration for each of the 78 SNP instrument. Association estimates derived directly from UK Biobank white British participants using BOLT-LMM.

File Name: Supplementary Data 3
Description: Association estimate for each of the 25(OH)D SNP instruments on cancer risk for each cancer type evaluated. Cancer traits obtained from the online MR-Base platform were excluded.

File Name: Supplementary Data 4
Description: Functional annotation for each of the 78 25(OH)D SNP instrument derived directly from PLINK v1.9b. The target gene was determined via location proximity (in kb). P-values refer to the SNP association p-value with rank-transformed 25(OH)D concentration.
